# Fast Intrinsic Emission Quenching in Cs_4_PbBr_6_ Nanocrystals

**DOI:** 10.1021/acs.nanolett.1c02537

**Published:** 2021-10-13

**Authors:** Urko Petralanda, Giulia Biffi, Simon C. Boehme, Dmitry Baranov, Roman Krahne, Liberato Manna, Ivan Infante

**Affiliations:** † Nanochemistry Department, Istituto Italiano di Tecnologia, Via Morego 30, 16163 Genova, Italy; ‡ Dipartimento di Chimica e Chimica Industriale, Università degli Studi di Genova, Via Dodecaneso, 31, 16146 Genova, Italy; § Department of Theoretical Chemistry, Faculty of Science, Vrije Universiteit Amsterdam, de Boelelaan 1083, 1081 HV Amsterdam, The Netherlands; # Optoelectronics Research Line, Istituto Italiano di Tecnologia, Via Morego 30, 16163 Genova, Italy

**Keywords:** Density Functional Theory, Green Emission, Nonradiative Quenching, Molecular Dynamics

## Abstract

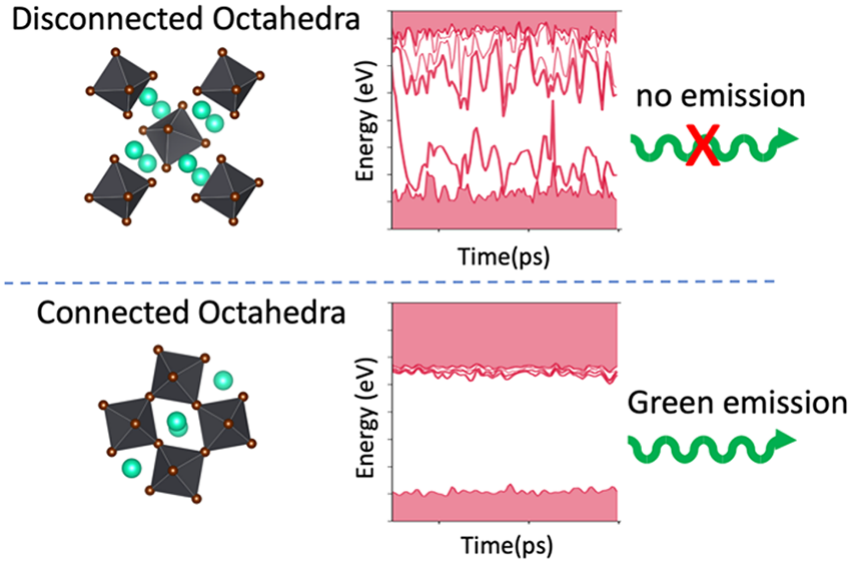

Cs_4_PbBr_6_ (0D) nanocrystals at room temperature have both been reported as nonemissive and green-emissive systems in conflicting reports, with no consensus regarding both the origin of the green emission and the emission quenching mechanism. Here, via ab initio molecular dynamics (AIMD) simulations and temperature-dependent photoluminescence (PL) spectroscopy, we show that the PL in these 0D metal halides is thermally quenched well below 300 K via strong electron–phonon coupling. To unravel the source of green emission reported for bulk 0D systems, we further study two previously suggested candidate green emitters: (i) a Br vacancy, which we demonstrate to present a strong thermal emission quenching at room temperature; (ii) an impurity, based on octahedral connectivity, that succeeds in suppressing nonradiative quenching via a reduced electron–phonon coupling in the corner-shared lead bromide octahedral network. These findings contribute to unveiling the mechanism behind the temperature-dependent PL in lead halide materials of different dimensionality.

Driven by the recent breakthroughs in lead halide perovskite (LHP) nanocrystals (NCs),^1−5^ their “zero-dimensional” lead bromide Cs_4_PbBr_6_ (0D) counterparts have gained attention in the last years.^6−9^ The lack of corner-sharing connectivity between the PbBr_6_ octahedra confers this 0D metal halide properties that are different from those of the intensively studied perovskites; the band structure of the bulk 0D system is mostly flat, and its electronic density is localized on each lead bromide unit in a molecule-like fashion. Consequently, the bandgap of the bulk 0D at room temperature of 3.95 eV is quasi-identical to that of a hypothetical isolated Cs_4_PbBr_6_ molecule.^10^ The formation of small and highly energetic polarons and self-trapped excitons^11−14^ enabled by such strong electronic localization makes 0D halides appealing from both fundamental and applicative standpoints.

At the core of an ongoing debate around 0D lead bromides is the origin of the green emission, frequently observed in bulk powders and single crystals and occasionally reported in nanocrystals (NCs).^15^ Early speculations of an intrinsic emission of the material^6,16,17^ gradually sedimented into two main lines of interpretation; i.e., the emission has been assigned to either deep band levels induced by point defects, likely Br vacancies,^16−21^ or to embedded nanoscale inclusions of, e.g., CsPbBr_3_ (3D)^7,12,22−27^ or lower-dimensional structures,^28^ whose small size evades facile characterization, e.g., via X-ray diffraction (XRD). Although more recent works lean toward the hypothesis of a 3D contamination,^29^ no final consensus has been achieved so far. This can be attributed to the difficulty of attaining an atomically resolved elemental mapping/structural analysis of crystals that would allow to unambiguously demonstrate the presence of a specific impurity or defect in the material. A way to overcome this experimental uncertainty is to use computational approaches.

In this work, we present an ab initio molecular dynamics (AIMD) study of bulk Cs_4_PbBr_6_ in a wide range of temperatures, from 34 K to room temperature (300 K).^30−36^ We demonstrate that strong electron–phonon coupling is responsible for fast nonradiative decay in the pure 0D structure already at temperatures well below 300 K. Ultimately, we discard the presence of point defects as a possible cause for the green emission of the crystal, due to a similar dynamic behavior as the pure crystal. Instead, we demonstrate that the connectivity between lead-bromide octahedra in the 3D structure suppresses nonradiative recombination, thus suggesting that the experimentally observed green emission is attributable to LHP-like impurities embedded in the 0D crystal. The results of our computational study are corroborated by spectroscopic experiments on 10 nm 0D NCs that are nonemissive at room temperature but become emissive at temperatures below ∼130 K.

## Computational PL Excitation and Emission Spectra of the 0D System

**Figure 1:**
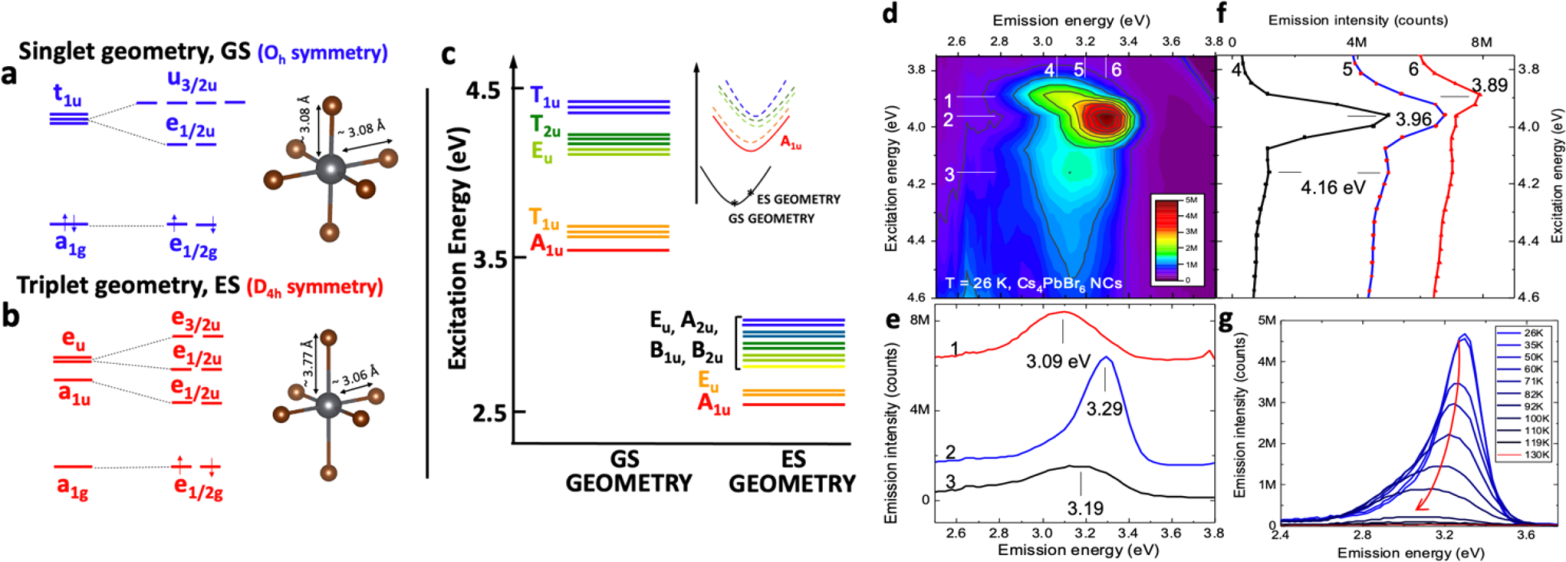
Computed energy levels of [Cs_8_PbBr_6_]^4+^ in the equilibrium geometries of the (a) singlet-spin-multiplicity state (GS) and (b) triplet-spin-multiplicity state (ES). Geometrical variations are highlighted in the structures. The energy levels are shown at the scalar relativistic level (left side) and upon inclusion of spin–orbit coupling (right side). (c) The 12 lowest-energy excitation energies computed at the TDDFT-SOC/PBE level of theory at the GS and ES geometries. (d) 2D excitation–emission map for the 0D NC sample at *T* = 26 K; (e) corresponding slices of emission spectra at three different excitation energies: 3.89, 3.96, and 4.16 eV (indicated by numbers 1, 2, and 3 in the 2D map, respectively); (f) corresponding slices of excitation spectra at the three emission maxima: 3.09, 3.19, and 3.29 eV (indicated by numbers 4, 5, and 6 in a 2D map, respectively); (g) temperature dependence of the most intense emission at 3.29 eV (excitation 3.96 eV), with spectra for two other peaks provided in the Supporting Information.

A robust computational methodology for structure, excitation, and emission characteristics of the defect-free 0D phase is an essential starting point (see Supporting Information for computational details). The band structure presents substantially flat conduction and valence bands,^10^ suggesting that the emissive centers are localized on the single octahedra. Because a full evaluation of spin–orbit coupling (SOC) on the entire supercell is computationally prohibitive, we decided to cleave a [Cs_8_PbBr_6_]^4+^ unit with a pseudo O_h_ symmetry from the supercell and compute the ground state and the lowest excited states at the ground state geometry (GS, singlet geometry, Figure 1a) and the excited state one (ES, triplet geometry, Figure 1b). The corresponding transition energies in the GS and ES geometries (Figure 1c) are in close agreement with the experimental PL excitation and emission spectra, respectively (Figure 1d,e). While the employed PBE functional leads to a slight underestimation of the computed band gap (∼3.6 eV) with respect to the experimental gap (∼3.95 eV, Figure 1f) and only qualitatively describes the oscillator strength,^33^ the computed Stokes shift of ∼1.0 eV between the lowest-energy excitation and emission state reproduces the experimental observations (see below). Furthermore, the calculated transition energies, oscillator strengths, radiative lifetimes, and symmetries of the 12 lowest-energy transitions (Table S1) serve as a basis for identifying and assigning the main features of the experimental excitation spectra.

## Experimental PL Excitation and Emission Spectra of 0D NCs

We investigated the optical properties of 0D NCs prepared by colloidal synthesis^37^ for a comparison with the calculations. These NCs are ∼10 nm in diameter and demonstrate a broad absorption peak at 5.44 eV and a narrow lowest-lying absorption peak at 3.95 eV in hexane dispersion (see Supporting Information). The 0D NCs are nonemissive in dispersion or drop-cast film at room temperature and show emission upon cooling below ∼130 K. Figure 1d shows a 2D excitation–emission map of the drop cast 0D NC sample measured at 26 K. The slices 1–3 along the emission energy axis reveal three PL peaks at 3.09, 3.29, and 3.19 eV (Figure 1e), while the slices 4–6 along the excitation axis reveal three absorption peaks at 4.16, 3.96, and 3.89 eV (Figure 1f). The 3.96 eV peak, the strongest feature in the excitation spectra, is virtually unchanged as compared to the lowest absorption peak at room temperature, is well-correlated with the most intense emission at 3.29 eV and is in line with the computed oscillator strengths in absorption that identify the second band (T_1u_ states) as the most optically active one.

## The Mechanism of PL Quenching

Figure 1g reports the temperature dependence of the most intense emission peak at 3.29 eV (Supporting Information for the temperature dependence of the other two). In all cases, an increase in temperature causes emission broadening and quenching at temperatures above 100 K. To provide further insights into the quenching mechanism, we performed AIMD simulations of the bulk Cs_4_PbBr_6_ at 34, 66, 110, and 300 K (Supporting Information for further details), i.e., in a temperature range similar to the experiment. The electronic structure of the system has been computed at each time step of the MD trajectory (2.5 fs) at the scalar relativistic DFT/PBE/DZVP level of theory on a 2 × 2 × 2 supercell. Due to the strong excitonic localization, consistent with the rather flat band structure of the 0D system,^10^ the k-sampling was limited to the Gamma point. Considering the size of the system and the fact that the PBE exchange-correlation functional accidentally provides a fair energy gap for lead–halide perovskite systems,^38^ we decided to describe ES by one-electron transitions.

**Figure 2:**
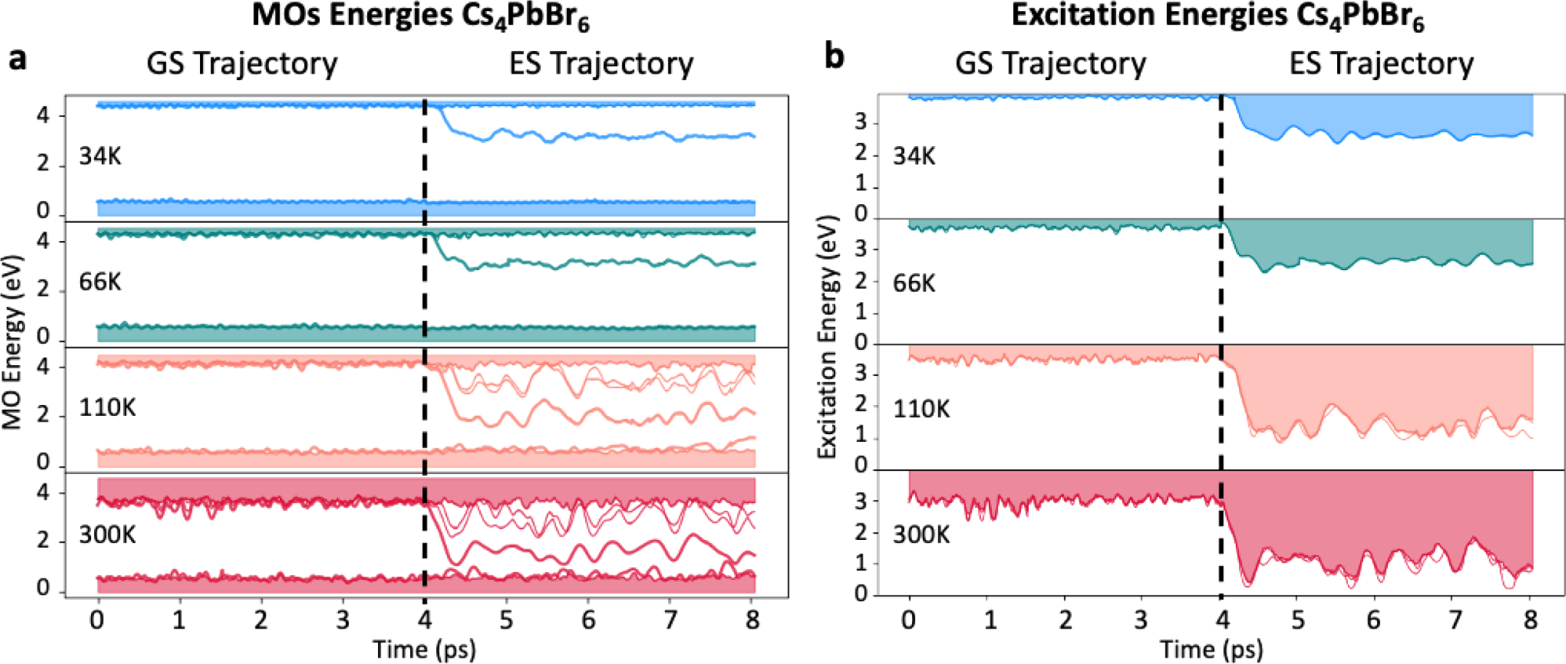
(a) Time evolution of the relevant electronic energy levels of Cs_4_PbBr_6_, calculated by ab initio MD at different temperatures. (b) Time evolution of the lowest three one-electron excitations computed as the energy difference between occupied and unoccupied molecular orbitals. The dotted line indicates the moment in which the system is excited from the ground state to the excited state, computed with the triplet spin configuration. All calculations are carried out at the DFT/PBE level of theory.

For temperatures from 34 to 300 K, Figure 2 shows the time-dependent energy fluctuations of relevant one-electron states during an AIMD trajectory of several picoseconds. The time evolution in the ground state (GS, singlet spin configuration without SOC, Figure 2a) is shown up to 4 ps (vertical dashed line), where a photoexcitation is emulated by continuing the AIMD trajectory for a triplet spin configuration. The latter configuration is chosen based on the triplet spin character of the lowest ESs. Since the triplet trajectory (i.e., *t* > 4 ps in Figure 2a) starts from the same structure and velocities as in the last point of the GS singlet trajectory, we here address, at least partially, also the effect of structural relaxation occurring right after photoexcitation.

To relate the observed energy-level fluctuations in GS and ES AIMD simulations to the experimentally observed optical bandgap in absorption and emission, Figure 2b displays the three lowest one-electron excitations, computed as energy differences between occupied and unoccupied molecular orbitals (MOs). While we should acknowledge that the depicted energy gaps in Figure 2b neglect possible many-body effects, we expect the latter to not change our results for this compound qualitatively.^31,32^ Consistently with the previous sections and prior reports,^39,40^ we obtain at 34 K an average band gap of ∼3.9 eV for GS, in excellent agreement with the measured absorption onset (3.95 eV).^40^ As expected, we notice minimal fluctuations of the gap throughout the simulation time, whereas when we steadily increase the temperature up to 300 K, the vibrational fluctuations increase and a clear nonuniformity of the gap appears, corresponding to a broadening of the first excitonic peak. We also observe a clear broadening as temperature increases, along with a slight redshift, in line with increased exciton–phonon coupling. Regarding the atomic positions, the occurrence of a phase transition implying a deformation of the isolated octahedra in Cs_4_PbBr_6_ could cause variations in its spectroscopic properties. However, previous Raman spectroscopy studies did not identify any phase transition of Cs_4_PbBr_6_ from room temperature down to 80 K.^29^

The triplet ES trajectory shows a relevant qualitative difference with the GS: after a few tens of femtoseconds, the lowest triply degenerate t_1u_ MOs (Figure 1a,b) are split significantly, reflecting the rupture of the octahedral symmetry upon photoexcitation due to Jahn–Teller distortion.^10^ Notice that, at low photon fluxes, only one exciton is formed, thus only one of the 48 octahedra is excited in our simulations. All other MOs remain unaffected and contribute equally to the GS. The Jahn–Teller distortion provokes a substantial reorganization, significantly decreasing the energy of unoccupied MOs, hereby decreasing the bandgap. Upon increasing the temperature from 34 to 300 K, time-dependent fluctuations of the gap increase in amplitude such that the energy difference between occupied and unoccupied MOs momentarily becomes very small. Its immediate implication is relevant: when the temperature is increased, many channels for nonradiative decay are opened, i.e., the unoccupied MOs collapse on the occupied ones. These channels are formed in a few picoseconds, especially at temperatures higher than 110 K. The destabilization of the ES is also evidenced by the difficult total energy (kinetic+potential) equilibration along the triplet MD trajectory right after excitation at 110 and 300 K.

This fast-quenching mechanism implies that the room temperature green emission of the emissive 0D crystals is not an intrinsic feature of the pure material but arises from other sources. This quenching mechanism is also in stark contrast with what is observed for the isostructural Cs_4_SnBr_6_ or Sb-doped Rb_3_InCl_6_, where the intrinsic photoemission is robust in a wide temperature range.^12,41,42^ In those systems, the exciton can survive quenching from high-temperature vibrational fluctuations despite similar reorganization energies as in Cs_4_PbBr_6_. On the other hand, the electron–phonon coupling in Cs_4_PbBr_6_ is much stronger in comparison to that of CsPbBr_3_, where the more efficient emission comes from excitons with lower binding energies (see below).

**Figure 3:**
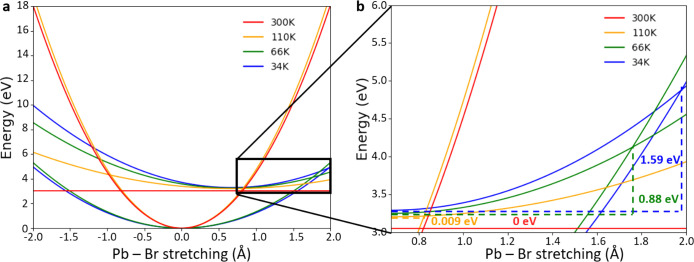
(a) One-dimensional parabolas constructed at different temperatures using the time-averaged energies from AIMD on the ground state (GS) and excited-state (ES) trajectories. The GS parabolas all intersect at the Pb–Br equilibrium position set as 0. The ES parabolas all intersect at the ES equilibrium position, stretched by 0.675 Å compared to the GS equilibrium distance. More details on how the parabolas are constructed are given in the Supporting Information. (b) Magnification of the black square in panel a, highlighting the variation of the energy of the ES minima and of its curvature. The reported values indicate the energy required to access a nonradiative decay path through the crossing with the respective GS curve for each temperature.

To obtain a more quantitative picture of the quenching mechanism, we use the time-averaged energies from the AIMD trajectories to construct a configuration coordinate diagram (Figure 3a). Herein, we approximate the potential-energy surfaces (PES) of the GS and ES at different temperatures with one-dimensional parabolas. The parabolas imply the adoption of the harmonic approximation along the main reaction coordinate that, in this case, is represented, for simplicity, by the axial Pb–Br bond, which is substantially elongated after photoexcitation. In this approximation, we consider all other changes, e.g., angle distortion and equatorial Pb–Br shrinkage, as minimal. In principle, within the harmonic approximation, the GS and ES parabola should be temperature independent, however, as it will be seen later, this is not strictly true because clear anharmonic effects take place at higher temperatures. To keep our model simple, however, we will employ a “coarse” harmonic approximation; i.e., the anharmonic effect is embedded still within a one-dimensional parabola, whose width changes with temperature.

The parabolas in Figure 3a are constructed using Nelsen’s four-point method, i.e. by assessing the energy at four different points along the PES.^43^ The first two points are taken at GS equilibrium Pb–Br bond distance, where we fix the lowest point from the average GS energy from the MD simulation, and the highest point from the average vertical HOMO–LUMO energy taken from the MD GS trajectory. The remaining two points are fixed at Pb–Br bond distance computed at ES optimized geometry, and whose energies are evaluated from the triplet ES trajectory (details in the Supporting Information). The parabolas allow us to estimate two parameters: the spring constants of the Pb–Br axial bond at the GS and ES geometries and the reorganization energies λ, i.e., the energy that the system needs to dissipate to reach the equilibrium geometry after the electronic transition has taken place (λ_0_ and λ_1_ in Table S3, for GS and ES, respectively). An alternative approach to construct the parabolas is presented in the Supporting Information and shows a similar qualitative behavior as above.

**Figure 4:**
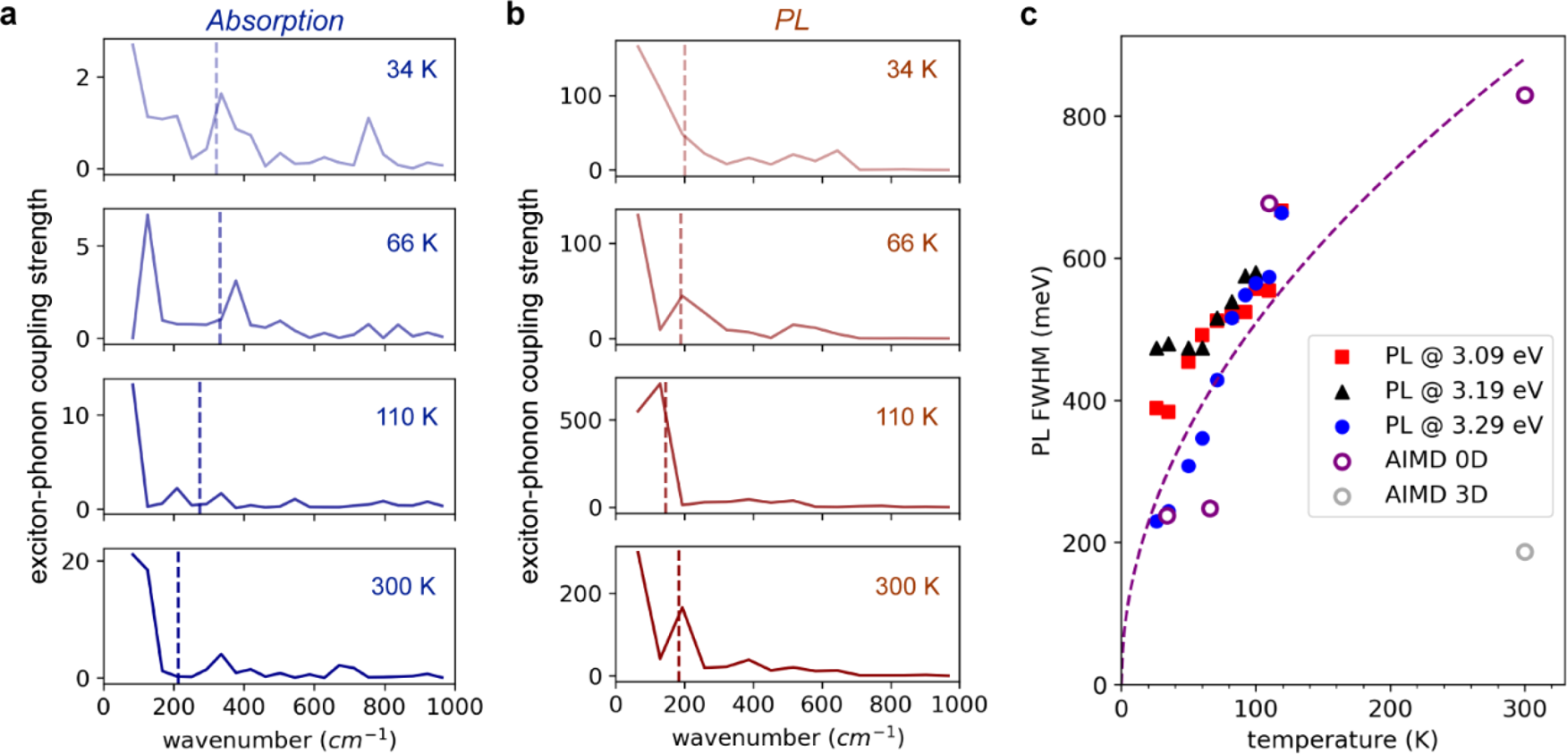
AIMD-derived temperature-dependent Huang–Rhys exciton–phonon coupling spectrum for Cs_4_PbBr_6_ in (a) absorption, and (b) PL. The dashed vertical lines indicate the average phonon energy, obtained after weighing all phonon modes with their corresponding coupling strength *S*. (c) Temperature-dependent PL fwhm in Cs_4_PbBr_6_ determined experimentally (solid symbols) for the PL bands displayed in Figure 1e, i.e., with maxima at 3.09 eV (red squares), 3.19 eV (black triangles), and 3.29 eV (blue circles), respectively, in good agreement with the PL fwhm computed from the AIMD trajectories (open purple circles). As a guide to the eye, a square-root dependence on temperature, as expected from a simple displaced harmonic-oscillator model, is depicted as a purple dashed line. For comparison, the computed room-temperature PL fwhm for 3D perovskites (CsPbBr_3_) is given as a gray open circle.

As anticipated, the obtained parabolas depicted in Figure 3a, differ, at the various temperatures, in curvature and in reorganization energy. Especially the variation in curvature strongly affects the energy and the reaction coordinate point where the GS and the ES curves cross. Employing Marcus theory,^44^ the nonradiative rate can be evaluated as
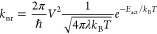
where *V* is the electronic coupling between the GS and ES estimated using the Mulliken–Hush approximation,^45^ and *E*_act_ is the activation energy for crossing nonradiatively from the ES PES to the GS PES (annotations in Figure 4b). Because the two parabolas present different curvatures, we assume that during the deactivation from the ES to GS only the GS rearranges, thus λ ∼ λ_GS_. Figure 3b shows that the activation energy for the nonradiative decay is reduced with increasing temperatures, suggesting accelerated nonradiative decay at room temperature. While we acknowledge that the rough approximation of translating GS and ES trajectories into one-dimensional parabolas can only be a coarse description of the real, potentially anharmonic PES, our configuration-coordinate diagram still provides a reasonable qualitative indication of the acceleration of the nonradiative decay with increasing temperature, in line with the temperature-dependent PL spectroscopy in Figure 1g.

To further investigate the phonon-induced nonradiative quenching mechanism, we quantify the coupling strength of phonons to electronic degrees of freedom by calculating the Huang–Rhys spectrum , where *ℏω* is the phonon energy,  denotes the fast Fourier transformation of the time-dependent energy difference *E*_*ij*_(*t*) between states *i* = HOMO and *j* = LUMO, *k*_B_ is the Boltzmann constant, and *T* the temperature. Figure 4a,b displays the computed Huang–Rhys spectra in absorption (using the energy gap fluctuations throughout the GS trajectory in Figure 2b) and in PL (using the energy gap fluctuations throughout the ES trajectory in Figure 2b), respectively. It is clear that many phonon modes couple to the HOMO–LUMO transition in both the absorption and emission processes. While the magnitude of the exciton–phonon coupling strength, *S*, is rather modest in absorption, in line with the experimentally found narrow PLE spectrum (Figure 1f), *S* increases by one to two orders of magnitude in emission, in line with the experimental finding of thermally quenched, broad, and red-shifted PL (see Figure 1g). Within the uncertainty of our AIMD-based computational methodology, we do not observe clear temperature-dependent trends for the Huang–Rhys coupling spectra. However, we notice a predominant coupling of low-energy phonons (≤200 cm^–1^) to the PL gap, consistent with the shallow PES in the ES (parabolas in Figure 3).

From the energy gap fluctuations of the ES trajectory (Figure 2b and related histograms in Figure S2), we can furthermore compute the expected PL fwhm for Cs_4_PbBr_6_. Figure 4c shows that the computed PL fwhm (purple open circles) is, at all temperatures, in excellent agreement with the experimentally determined one for this material (solid markers, obtained from the PL spectra in Figure 1g). These results lead for several conclusions: (a) the good agreement between experiment and theory lends credibility to the employed computational framework; (b) the similarity to a proportionality to the square root of the temperature (dashed line in Figure 4c) is consistent with the expected temperature dependence for the simple displaced harmonic-oscillator model employed to deduce the nonradiative quenching rates (Figure 3); (c) the more than four times larger PL fwhm in Cs_4_PbBr_6_ compared to CsPbBr_3_ (gray open circle) is a clear manifestation of the large exciton–phonon coupling in Cs_4_PbBr_6_. Overall, we suggest that the significant exciton–phonon coupling in Cs_4_PbBr_6_ is a key factor to include when explaining the experimentally observed thermally activated nonradiative PL quenching in this material.

## Origin of Green Emission

After clarifying the intrinsic features of the material, we use this approach as a predictive methodology to unravel the origin of the green emission in 0D crystals. To this aim, we decided to model the two systems that are considered^9,10^ responsible for the green emission: a defective 0D with a Br vacancy and a pure 3D CsPbBr_3_ inclusion mimicking the effect of interconnecting lead bromide octahedra. Similarly to the pure material, we performed room-temperature simulations of the GS and ES of both systems (details in the Supporting Information). For the Br vacancy, we modeled the same 2 × 2 × 2 supercell of the 0D phase calculations where we removed a CsBr atom pair to keep charge neutrality. For the 3D, we employed a 2 × 2 × 2 orthorhombic CsPbBr_3_ supercell.

**Figure 5:**
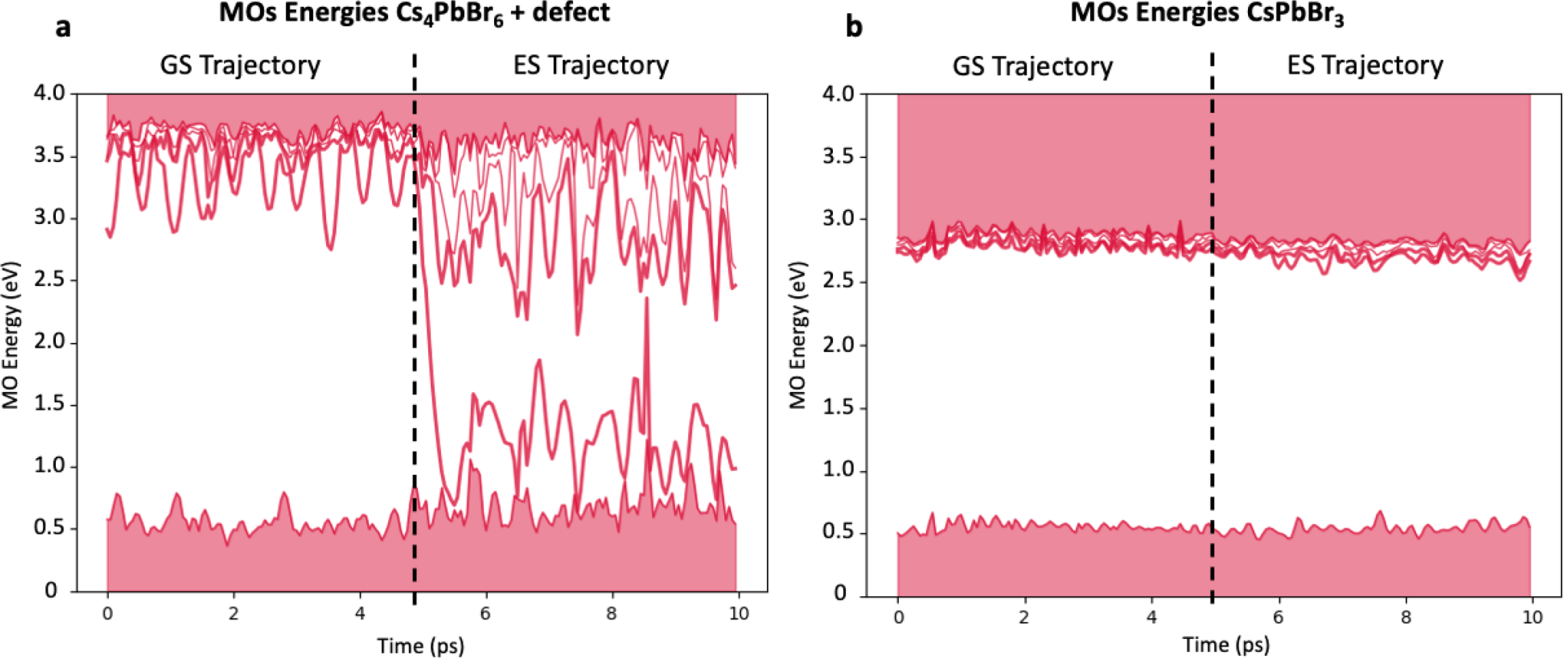
Time evolution of relevant electronic energy levels at 300 K calculated by AIMD for (a) CsPbBr_3_ with a CsBr pair vacancy and (b) Cs_4_PbBr_6_. Both plots contain the AIMD results for the respective GS (left of the dashed line) and ES (right of the dashed line).

Figure 5a shows the results obtained for the Br vacancy system. In the GS trajectory, we observe a slight perturbation on the electronic structure levels of the pure 0D material, where the LUMOs vibrational fluctuations are significant and oscillate even more widely than in the pure 0D. In the ES simulations, however, we see wide fluctuations of the electronic levels through the simulations at room temperature that point toward a thermal quenching of any emission that could be originated from Br vacancies, as in the case of the pure 0D crystal.

In the case of the 3D perovskite (Figure 5b), we find a well-defined band gap of around 2 eV, in line with the experimental value, which changes very little switching from the GS to the ES trajectory, consistent with the minimal Stokes shift typically found in 3D perovskites. Even at 300 K, phonon-induced energy-level fluctuations are far smaller than the band gap, such that no crossing between energy levels of occupied and unoccupied MOs are observed throughout the entire AIMD trajectory. Hence, we can deduce a very long time scale for nonradiative recombination, which likely cannot compete with the rapid radiative recombination in 3D perovskites. From the simulations, it is then evident that a 3D defect embedded in the 0D crystal, or any other defect that connects the octahedra, will remain emissive regardless of the vibrational fluctuations. This, in contrast with the high band crossing amount still taking place with the Br vacancy system, identifies the impurities with octahedra interconnection as the only reasonable source of green emission in 0D lead bromides at room temperature.

## Supporting Information Available

All calculated oscillator strengths; the technical details of the AIMD calculations; details of the methodology employed to construct the energy parabolas in Figure 3 and to derive the PL fwhm from the time-dependent energy-gap fluctuations in AIMD; details on the synthesis, characterization, and spectroscopic experiments on Cs_4_PbBr_6_ nanocrystals; additional temperature-dependent emission spectra (PDF)
