# Supplementary material for: Fast Intrinsic Emission Quenching in Cs4PbBr6 Nanocrystals
Source: Nano Lett. 2021 Oct 13;21(20):8619–26. doi: 10.1021/acs.nanolett.1c02537 (PMC8554796; doi:10.1021/acs.nanolett.1c02537)
Supplement: Supplementary file 1 — nl1c02537_si_001.pdf [file nl1c02537_si_001.pdf]

Supporting Information for

Fast Intrinsic Emission Quenching in Cs_4PbBr_6 Nanocrystals

Urko Petralanda,[†‡] Giulia Biffi,[Δ†‡], Simon C. Boehme,[§] Dmitry Baranov,[†] Roman Krahne,[†] Liberato Manna,[†*] Ivan Infante[†§*]

[†]Nanochemistry Department, Istituto Italiano di Tecnologia, Via Morego 30, 16163 Genova, Italy

[Δ] Dipartimento di Chimica e Chimica Industriale, Università degli Studi di Genova, Via Dodecaneso, 31, 16146 Genova, Italy

[§]Department of Theoretical Chemistry, Faculty of Science, Vrije Universiteit Amsterdam, de Boelelaan 1083, 1081 HV Amsterdam, The Netherlands

Present Addresses

Urko Petralanda current address is:

CAMD, Department of Physics, Technical University of Denmark, 2800 Kongens Lyngby, Denmark

Simon C. Boehme current affiliations are:

Institute of Inorganic Chemistry, Department of Chemistry and Applied Bioscience, ETH Zürich, 8093 Zürich, Switzerland

Laboratory for Thin Films and Photovoltaics, Empa – Swiss Federal Laboratories for Materials Science and Technology, 8600 Dübendorf, Switzerland

Section S1. Calculations on single octahedra. A single $[Cs_8PbBr_6]^{4+}$ octahedral unit with a pseudo O_h symmetry was cleft from the relaxed 2x2x2 supercells of the Cs_4PbBr_6 at the relaxed ground state (GS) (closed shell spin configuration) and excited state (ES) geometries (triplet spin configuration). The optical transitions (Table S1) were computed at the TDDFT/PBE/DZVP as implemented in the ADF2020 software[1–5]. Spin-orbit coupling was included from the outset. In the spin-free case, excitation occurs from the highest occupied molecular orbital (HOMO), localized on the antibonding 6s(Pb)-4p(Br) molecular orbital (MO), into the triply degenerate t_{1u} lowest unoccupied MOs (LUMOs), localized on the antibonding 6p(Pb)-4p(Br) MOs, giving origin to the three degenerate excited states (Figure 1a of the main text). In the presence of SOC, the LUMO splits into $e_{1/2u}$ and $u_{3/2u}$ contributions (Figure 1a) and 12 excitations are now possible that mix triplet and singlet spin configurations. In the isolated 0D species, SOC is very important because it produces a substantial split in the energy levels that greatly facilitates the interpretation of the experimental absorption and excitation spectra. Additionally, typical spin-forbidden transitions become optically allowed with values of the oscillator strength that can be qualitatively associated to the observed lifetimes of radiative recombination in time-resolved PL. The computed absorption spectrum at the GS geometry is depicted in Figure 1c of the main text. We can notice that the lowest 9 excited states have dominant triplet character and thus a low oscillator strength (Table S1). Four bands are discernible, with the first composed of the lowest A_{1u}, which is almost completely dark; then one composed of T_{1u} states, at 3.65 eV (f=~0.01), another from the E_u and T_{2u} states at 4.25 eV (f=0.0001) and the latter made of T_{1u} at 4.32 eV (f=0.01).

As expected, a mixing in the spin character of the states is obtained although a singlet-triplet nature is still detectable. We can still discern the dominant character (singlet or triplet) in the values of the oscillator strength and the lifetimes associated to each transition: the lowest excited state is almost completely dark, and the three states above are degenerate and among the brightest. From this analysis we can reasonably consider the lowest excited state with a dominant a triplet character.

Energy (eV)	Oscillator strength	τ (s)	Symmetry
3.52	2.8×10^{-9}	0.656	A_{1u}
3.64	1.7×10^{-2}	1.0×10^{-7}	T_{1u}
3.65	1.7×10^{-2}	1.0×10^{-7}	T_{1u}
3.66	1.8×10^{-2}	$9.8 \times 10\text{-}^{8}$	$T1_u$
4.24	9.1×10^{-5}	1.4×10^{-5}	E_u
4.24	9.2×10^{-5}	1.4×10^{-5}	E_u
4.24	3.3×10^{-7}	3.9×10^{-3}	T_{2u}

4.25	2.6×10^{-6}	4.9×10^{-4}	T_{2u}
4.25	2.6×10^{-6}	4.9×10^{-4}	T_{2u}
4.31	1.1×10^{-2}	1.2×10^{-6}	T_{1u}
4.34	1.1×10^{-2}	1.2×10^{-6}	T_{1u}
4.34	1.1×10^{-2}	1.1×10^{-6}	T_{1u}

Table S1. Lowest 12 excitations energies taking place in the $[Cs_8PbBr_6]^{4+}$ unit at the GS equilibrium geometry of the 2x2x2 0D supercell. Symmetries have been assigned on the basis of calculations performed with enforced O_h point group symmetry.

We have also optimized the triplet state geometry and performed the same analysis in these new nuclear coordinates. The relaxed structure features one of the octahedra with an axial Pb-Br bond stretching of 0.675 Å, due to the population of the antibonding 6p(Pb)-4p(Br) MOs, t1u orbitals, as observed previously in other works6 , and as expected for self-trapped excitons. This elongation strongly modifies the symmetry of the isolated octahedron, which now belongs to a pseudo D4h point group. The inspection of the lowest 12 excited states of the $[Cs_8PbBr_6]^{4+}$ unit at the ES geometry reveals a strong rearrangement of the energy spectrum (Figure 1c of the main text, ES), which now presents only two main bands, one composed of three almost degenerate low-energy states (at about 2.5 eV) and a second one, broader, with nine states all lying between 2.73-2.80 eV. The computed properties of these states are reported in Table S2. Considering that the total symmetry is not purely D4h, it is extremely difficult to assign a symmetry label to all the excited states.

Energy (eV)	Oscillator strength	τ (s)	Symmetry
2.53	1.7×10^{-8}	0.212	A_{1u}
2.55	2.3×10^{-3}	1.5×10^{-6}	E_u
2.55	2.4×10^{-3}	1.5×10^{-6}	E_u
2.73	1.4×10^{-4}	2.2×10^{-5}	
2.74	1.2×10^{-3}	2.5×10^{-6}	
2.74	1.5×10^{-3}	2.0×10^{-6}	
2.78	3.8×10^{-4}	7.8×10^{-6}	
2.78	6.0×10^{-4}	5.0×10^{-6}	
2.79	1.3×10^{-4}	2.3×10^{-5}	
2.79	2.1×10^{-3}	1.4×10^{-6}	
2.80	2.2×10^{-3}	1.4×10^{-6}	
2.80	2.3×10^{-3}	1.3×10^{-6}	

Table S2. Lowest 12 excitation energies taking place in $[Cs_8PbBr_6]^{4+}$ unit at the GS equilibrium geometry of the 2x2x2 0D supercell.

Section S2. Molecular dynamics and single point calculations. The supercell described in the main text was used to compute the molecular dynamics trajectories at 300K, 110K, 66K and 34K within an NPT ensemble. Each dynamics featured 5000 steps with a timestep of 2.5 fs, using a canonical velocity-rescaling algorithm. The trajectories in the triplet excited states used the last point of the GS trajectory as a starting point. In all cases, the PBE exchange-correlation functional,[7] Goedecker-Teter-Hutter pseudopotentials[8] and the MOLOPT-DZVP basis set[4] were employed within the mixed Gaussian and plane waves (GPW) formalism. The Cp2k 6.1 package was used for all calculations.[9–14] The calculations were planned and performed using the QMFlows package.[15]

Section S3. Construction of one-dimensional parabolas. The one-dimensional parabolas plotted in the main text were obtained using the 4-point method (see Figure S1a,b), in which point 1 and 3 are computed by averaging total GS and ES single-point energies computed in the last 2000 frames of the GS and ES trajectories (SP in Figure S1a), respectively, of the 2x2x2 0D supercell. Point 2 is computed by averaging the HOMO-LUMO gap energies from the single point GS calculations (H-L in Figure S1a). Point 4 instead is computed by averaging the differences between the energies of the single point ES calculations and the corresponding HOMO-LUMO energies (3 – H-L in Figure S1a). The shift along the reaction coordinate is taken by the stretch of the Pb-Br bond from the GS to ES optimized configurations.

This 4-point approach was chosen for a matter of consistency throughout the text, where the HOMO-LUMO energies are always employed. However, a more accurate way to reconstruct these curves is to perform single point total energy calculations for both the GS and the ES at both equilibrium geometries, thus obtaining total energies for each of the four points. Figure S1c,d show the comparison between the curves obtained with the two different approaches and Table S3 reports the various parameters obtained entering the Marcus equations. Indeed, the choice of these coordinates allows us to obtain the reorganization energies, i.e. the energy that the system needs to dissipate to reach the equilibrium geometry after the electronic transition has taken place (λ_{GS} and λ_{ES}, for GS and ES respectively). For a given approach, the parabolas differ in curvature (k_{GS} and k_{ES} in Table S3, for GS and ES respectively) and in the ground to excited state minima energy difference (ΔE_{minima}). Especially the variation in curvature strongly affects the activation energy and the reaction coordinate point at which the ground state and the excited state curves cross. As a matter of comparison, Table S3 reports also the properties

computed at 0K, i.e. without MD but only obtained from the geometry optimization of the four aforementioned points. Being the second approach more accurate, it provides quantitatively more correct parameters, but the qualitative results agree as an almost barrierless non-radiative recombination is obtained at room temperature, thus causing immediate quenching of the luminescence. A bit of disagreement arises at 110K, where the HOMO-LUMO approach predicts an extremely low barrier, which would correspond again to a very fast quenching, while from the four points approach the barrier is larger, in good agreement with the presence of a low-intensity yet well-defined peak in photoluminescence data.

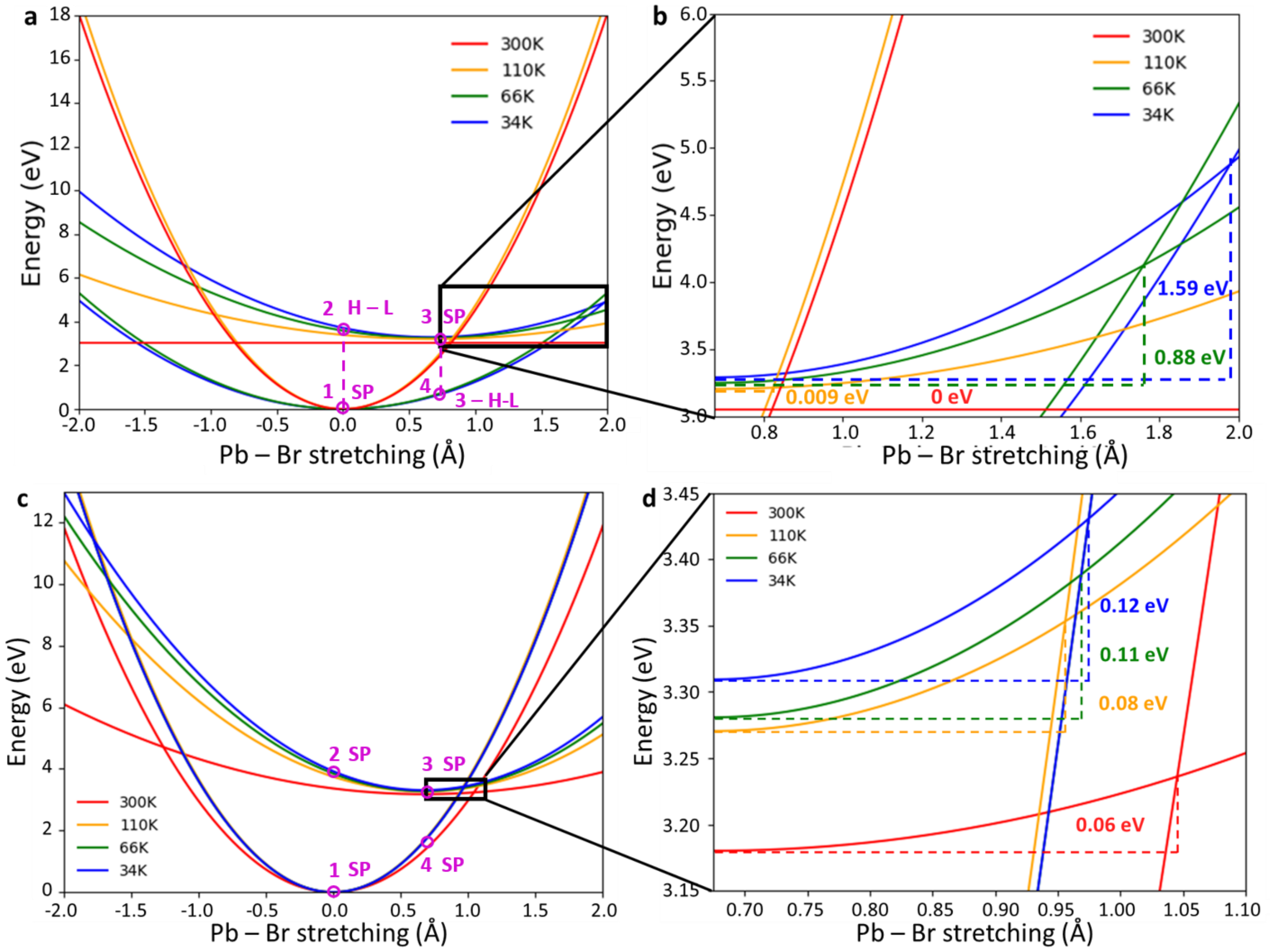

Figure S1: a) Parabolas computed using averaged GS single-point (SP) energies and HOMO-LUMO (H-L) energies and b) magnification at the minimum of the ES curve. c) Parabolas computed using averaged single-point energies in both the equilibrium geometries and both spin states and d) magnification at the minimum of the ES curve.

	4-points from HOMO-LUMO energies				4-points from total energies				
	300K	110K	66K	34K	300K	110K	66K	34K	0K
ΔE_{minima} (eV)	3.05	3.2	3.25	3.29	3.180	3.270	3.281	3.309	3.454
λ_{GS} (eV)	2.06	2.16	0.61	0.57	1.349	1.671	1.645	1.645	0.815
λ_{ES} (eV)	0	0.19	0.34	0.43	0.186	0.477	0.568	0.615	0.735
k_{GS} (eV/Å^2)	9.05	9.47	2.66	2.49	5.921	7.336	7.220	7.220	3.583
k_{ES} (eV/Å^2)	0	0.83	1.49	1.87	0.815	2.095	2.495	2.700	3.225
E_{act} (eV)	0	0.01	0.88	1.59	0.056	0.083	0.108	0.121	1.661

Table S3. Parameters extracted from the construction of the GS and ES parabolas using the time averaged AIMD results at the various temperatures. ΔE_{minima} identifies the GS to ES minima energy difference, k_{GS} and k_{ES} represent the spring constants (and are thus related to the curvatures) of the GS and ES parabolas, respectively. λ_{GS} and λ_{ES} are the reorganization energies for GS and ES respectively, i.e. the energy that needs to be dissipated to reach the equilibrium geometry after an electronic transition has taken place, whereas E_{act} is the energy required to overcome the barrier from the ES minimum to the crossing point with the GS curve in order to obtain non-radiative decay. The last column indicates results obtained from parabolas reconstructed using single point DFT calculations performed at 0K.

Section S4. Derivation of the PL FWHM from the time-dependent energy-gap fluctuations in AIMD. The left column in Figure S2 displays the time-dependent energy-gap fluctuations associated to absorption (red traces) and PL (blue traces) in Cs_4PbBr_6. Gaussian fits to the histograms (see right column of Figure S2) yield the FWHM of both absorption and PL. The thus obtained PL FWHM is plotted in Figure 4b of the main text.

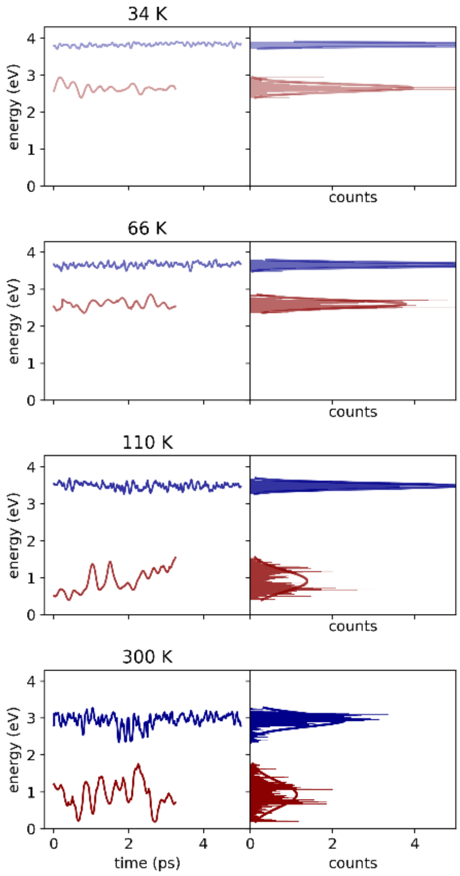

Figure S2. AIMD-derived PL FWHM in Cs_4PbBr_6. *Left column*: time-dependent energy gap fluctuations in absorption (red traces, obtained from the gaps in the GS trajectory of Figure 2b) and PL (blue traces, obtained from the gaps in the ES trajectory of Figure 2b), respectively. *Right column:* histograms (horizontal bars) and associated Gaussian fits (thick solid lines) for both the absorption and PL transitions. The FWHM of the Gaussian fits to the PL histograms is plotted in Figure 4b of the main text.

Section S5. Nanocrystal synthesis, characterization, and temperature-dependent spectroscopy.

Nanocrystals of Cs_4PbBr_6 were synthesized by injecting cesium oleate into the solution of lead(II) bromide in 1-octadecene and oleic acid, and oleylamine, following previously established procedure (see Section S1 in the Supporting Information of Ref. 16 for details). Post-synthesis, the nanocrystals were isolated by centrifugation without addition of anti-solvent and subsequently redispersed in hexane yielding a clear and colorless dispersion.

The produced nanocrystals have Cs-rich and Br-poor composition (Cs:Pb:Br ≈ 4.8 : 1 : 5.4) and consist of crystalline cores (rhombohedral Cs_4PbBr_6, ICSD 162158)[17] capped with a mixture of ligands (cesium oleate, oleylammonium oleate, and neutral oleylamine) with a total oleylammonium oleate to neutral oleylamine ratio of ~3:2, as determined by a combination of elemental analysis, X-ray diffraction, and Nuclear Magnetic Resonance spectroscopy in the preceding work.[16] For comparison with tin-based compound of the same formula, the rhombohedral Cs_4PbBr_6 is isostructural with Cs_4SnBr_6 (ICSD 434641)[18] and belong to the same space group ($R\bar{3}c$, space group number 167). A recent work by Chen et al. studied powders of bulk Cs-rich (Cs:Pb:Br = 4.1 : 1 : 6.1) and Pb-rich (Cs:Pb:Br = 3.5 : 1 : 5.0) Cs_4PbBr_6 compounds and found both types of samples to be green-emissive due to the impurity of CsPbBr3 perovskite.[19] In other work, Qin et al. synthesized non-emissive Cs4PbBr6 nanocrystals using synthetic protocol very similar to ours and compared them with green-emissive Cs4PbBr6 nanocrystals synthesized by precipitation method at room temperature.[20] No elemental composition of green-emissive and non-emissive Cs_4PbBr_6 nanocrystals has been reported in that work, but a comparison of Raman spectra at T= 80 K showed that green-emissive Cs_4PbBr_6 nanocrystals contained an additional Raman band at ~29 cm-1 that matched a band in the Raman spectrum of perovskite CsPbBr3 nanocrystals, hence again presenting an evidence that the perovskite CsPbBr3 compound is a source of the green emission. Overall, the lack of green emission in our nanocrystals (Figure S3) is consistent with the interpretation that studied nanocrystals are perovskite-free and the deviation from ideal stoichiometry is caused by surface termination with cesium oleate.

A small amount of the nanocrystal dispersion was drop-cast on a sapphire substrate for optical experiments. Figure S4 shows optical absorption of the hexane dispersion of ~10 nm Cs_4PbBr_6 nanocrystals (inset shows a transmission electron microscopy image of the nanocrystal sample) and an attempted measurement of the photoluminescence spectrum of the nanocrystal thin film that yielded no detectable emission at room temperature (T = 296 K). Subsequently, the sapphire substrate with a nanocrystal film was placed inside a

closed-cycle helium cryostat (Advanced Research Systems, DE204SI) and the photoluminescence and photoluminescence excitation spectra were recorded using Edinburgh FLS920 spectrofluorimeter equipped with a Xe lamp (Xe900, an excitation source) and optical fibers for sample excitation and signal collection.

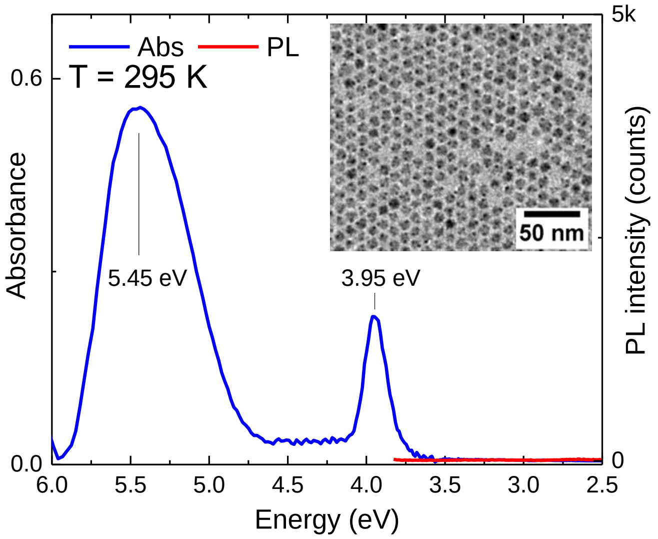

Figure S3. Room temperature absorption spectrum of hexane dispersion of ~10 nm Cs_4PbBr_6 nanocrystals (blue solid line) and an attempted photoluminescence spectrum of drop cast film excited at 3.95 eV (red solid line) demonstrating no detectable emission at room temperature. The inset shows a low magnification transmission electron microscopy image of the nanocrystal sample.

Figure S4 shows the temperature-dependent PL plots of the three peaks.

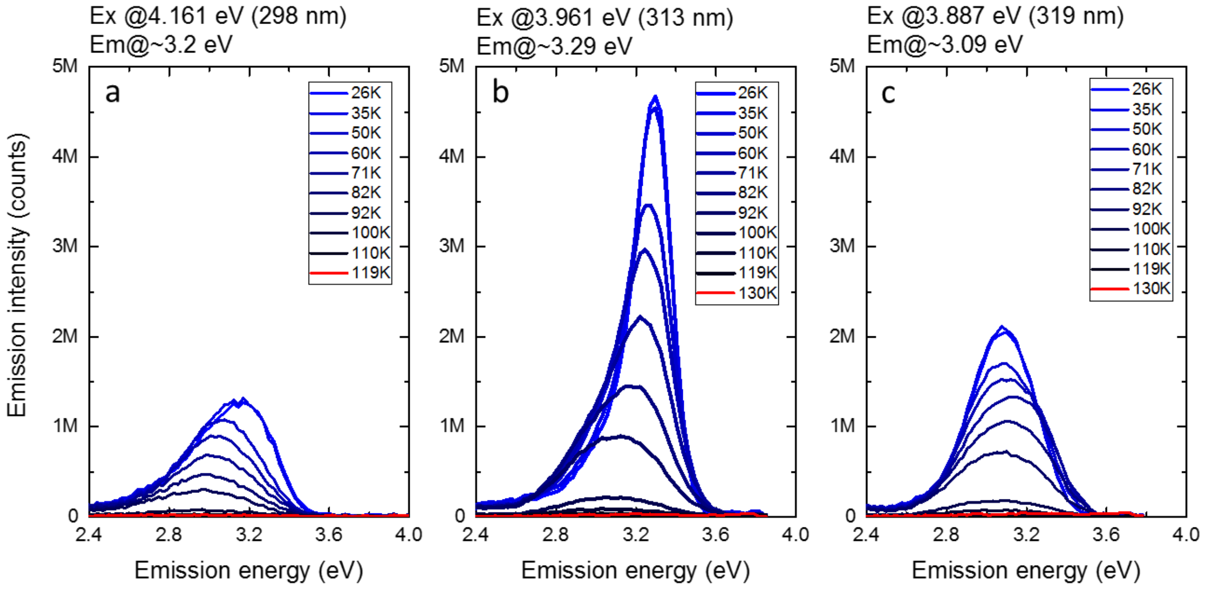

Figure S4. The temperature dependence of the three emission peaks observed in the thin film sample of Cs_4PbBr_6 nanocrystals and shown in Figure 1d in the main text. a) The emission centred at ~3.2 eV (excitation at 298 nm), b) the strongest emission centred at ~3.29 eV (excitation at 313 nm), and c) the emission centred at ~3.09 eV (excitation 319 nm).

REFERENCES

(1) te Velde, G.; Bickelhaupt, F. M.; Baerends, E. J.; Fonseca Guerra, C.; van Gisbergen, S. J. A.; Snijders, J. G.; Ziegler, T. Chemistry with ADF. *J. Comput. Chem.* **2001**, *22* (9), 931–967. https://doi.org/10.1002/jcc.1056.

(2) Versluis, L.; Ziegler, T. The Determination of Molecular Structures by Density Functional Theory. The Evaluation of Analytical Energy Gradients by Numerical Integration. *J. Chem. Phys.* **1988**, *88* (1), 322–328. https://doi.org/10.1063/1.454603.

(3) Fan, L.; Ziegler, T. Nonlocal Density Functional Theory as a Practical Tool in Calculations on Transition States and Activation Energies. Applications to Elementary Reaction Steps in Organic Chemistry. *J. Am. Chem. Soc.* **1992**, *114* (27), 10890–10897. https://doi.org/10.1021/ja00053a027.

(4) VandeVondele, J.; Hutter, J. Gaussian Basis Sets for Accurate Calculations on Molecular Systems in Gas and Condensed Phases. *J. Chem. Phys.* **2007**, *127* (11), 114105. https://doi.org/10.1063/1.2770708.

(5) van Gisbergen, S. J. A.; Snijders, J. G.; Baerends, E. J. Implementation of Time-Dependent Density Functional Response Equations. *Comput. Phys. Commun.* **1999**, *118* (2), 119–138. https://doi.org/https://doi.org/10.1016/S0010-4655(99)00187-3.

(6) Yin, J.; Maity, P.; De Bastiani, M.; Dursun, I.; Bakr, O. M.; Bredas, J.-L.; Mohammed, O. F. Molecular Behavior of Zero-Dimensional Perovskites. *Sci. Adv.* **2017**, *3* (12). https://doi.org/10.1126/sciadv.1701793.

(7) Perdew, J. P.; Burke, K.; Ernzerhof, M. Generalized Gradient Approximation Made Simple. *Phys. Rev. Lett.* **1996**, *77* (18), 3865–3868. https://doi.org/10.1103/PhysRevLett.77.3865.

(8) Krack, M. Pseudopotentials for H to Kr Optimized for Gradient-Corrected Exchange-Correlation Functionals. *Theor. Chem. Acc.* **2005**, *114* (1), 145–152. https://doi.org/10.1007/s00214-005-0655-y.

(9) Kühne, T. D.; Iannuzzi, M.; Del Ben, M.; Rybkin, V. V; Seewald, P.; Stein, F.; Laino, T.; Khaliullin, R. Z.; Schütt, O.; Schiffmann, F.; Golze, D.; Wilhelm, J.; Chulkov, S.; Bani-Hashemian, M. H.; Weber, V.; Borštnik, U.; Taillefumier, M.; Jakobovits, A. S.; Lazzaro, A.; Pabst, H.; Müller, T.; Schade, R.; Guidon, M.; Andermatt, S.; Holmberg, N.; Schenter, G. K.; Hehn, A.; Bussy, A.; Belleflamme, F.; Tabacchi, G.; Glöß, A.; Lass, M.; Bethune, I.; Mundy, C. J.; Plessl, C.; Watkins, M.; VandeVondele, J.; Krack, M.; Hutter, J. CP2K: An Electronic Structure and Molecular Dynamics Software Package - Quickstep: Efficient and Accurate Electronic Structure Calculations. *J. Chem. Phys.* **2020**, *152* (19), 194103. https://doi.org/10.1063/5.0007045.

(10) Schütt, O.; Messmer, P.; Hutter, J.; VandeVondele, J. GPU-Accelerated Sparse Matrix–Matrix Multiplication for Linear Scaling Density Functional Theory. *Electronic Structure Calculations on Graphics Processing Units*. March 2016, pp 173–190. https://doi.org/https://doi.org/10.1002/9781118670712.ch8.

(11) Borštnik, U.; VandeVondele, J.; Weber, V.; Hutter, J. Sparse Matrix Multiplication: The Distributed Block-Compressed Sparse Row Library. *Parallel Comput.* **2014**, *40* (5), 47–58. https://doi.org/https://doi.org/10.1016/j.parco.2014.03.012.

(12) Hutter, J.; Iannuzzi, M.; Schiffmann, F.; Vandevondele, J. Cp2k: Atomistic Simulations of Condensed Matter Systems. *Wiley Interdiscip. Rev. Comput. Mol. Sci.* **2014**, *4* (1), 15–25. https://doi.org/10.1002/wcms.1159.

(13) Bussi, G.; Donadio, D.; Parrinello, M. Canonical Sampling through Velocity Rescaling. *J. Chem. Phys.* **2007**, *126* (1), 014101. https://doi.org/10.1063/1.2408420.

(14) VandeVondele, J.; Krack, M.; Mohamed, F.; Parrinello, M.; Chassaing, T.; Hutter, J. Quickstep: Fast and Accurate Density Functional Calculations Using a Mixed Gaussian and Plane Waves Approach. *Comput. Phys. Commun.* **2005**, *167* (2), 103–128. https://doi.org/https://doi.org/10.1016/j.cpc.2004.12.014.

(15) Zapata, F.; Ridder, L.; Hidding, J.; Jacob, C.; Infante, I.; Visscher, L. QMflows: A Tool Kit for Interoperable Parallel Workflows in Quantum Chemistry. *J. Chem. Inf. Model.* *59* (7), 3191–3197. https://doi.org/10.1021/acs.jcim.9b00384.

(16) Baranov, D.; Caputo, G.; Goldoni, L.; Dang, Z.; Scarfiello, R.; De Trizio, L.; Portone, A.; Fabbri, F.; Camposeo, A.; Pisignano, D.; Manna, L. Transforming Colloidal Cs_4PbBr_6 Nanocrystals with Poly(Maleic Anhydride-Alt-1-Octadecene) into Stable $CsPbBr_3$ Perovskite Emitters through Intermediate Heterostructures. *Chem. Sci.* **2020**, *11* (15), 3986–3995. https://doi.org/10.1039/d0sc00738b.

(17) Velázquez, M.; Ferrier, A.; Péchev, S.; Gravereau, P.; Chaminade, J.-P.; Portier, X.; Moncorgé, R. Growth and Characterization of Pure and Pr^{3+}-Doped Cs_4PbBr_6 Crystals. *J. Cryst. Growth* **2008**, *310* (24), 5458–5463. https://doi.org/10.1016/j.jcrysgro.2008.10.003.

(18) Benin, B. M.; Dirin, D. N.; Morad, V.; Wörle, M.; Yakunin, S.; Rainò, G.; Nazarenko, O.; Fischer, M.; Infante, I.; Kovalenko, M. V. Highly Emissive Self-Trapped Excitons in Fully Inorganic Zero-Dimensional Tin Halides. *Angew. Chemie Int. Ed.* **2018**, *57* (35), 11329–11333. https://doi.org/10.1002/anie.201806452.

(19) Chen, X.; He, M.; Huang, S.; Zhang, L.; Zhong, H. Interlayer Determined Photoluminescence Excitation Properties of Cs-Rich and Pb-Rich Cs_4PbBr_6 Samples. *J. Phys. Chem. C* **2021**, *125* (29), 16103–16109. https://doi.org/10.1021/acs.jpcc.1c01908.

(20) Qin, Z.; Dai, S.; Hadjiev, V. G.; Wang, C.; Xie, L.; Ni, Y.; Wu, C.; Yang, G.; Chen, S.; Deng, L.; Yu, Q.; Feng, G.; Wang, Z. M.; Bao, J. Revealing the Origin of Luminescence Center in 0D Cs_4PbBr_6 Perovskite. *Chem. Mater.* **2019**, *31* (21), 9098–9104. https://doi.org/10.1021/acs.chemmater.9b03426.
